# Expansion of human cord blood hematopoietic stem/progenitor cells in three-dimensional Nanoscaffold coated with Fibronectin

**Published:** 2015-04-01

**Authors:** Seyed Hadi Mousavi, Saeid Abroun, Masoud Soleimani, Seyed Javad Mowla

**Affiliations:** 1 PhD student of Hematology, Department of Hematology and Blood Banking, Faculty of Medical Sciences, Tarbiat Modares University, Tehran, Iran; 2 Associated Professor of Hematology, Department of Hematology and Blood Banking, Faculty of Medical Sciences, Tarbiat Modares University, Tehran, Iran; 3 Associated Professor of Genetic, Department of Molecular Genetics, Faculty of Biological Sciences, Tarbiat Modares University, Tehran, Iran

**Keywords:** Cord Blood Stem Cell Transplantation, Hematopoietic Stem Cells, Tissue Engineering, 3D culture

## Abstract

**Background: **Allogeneic hematopoietic stem cell transplantation is used in the treatment of patients suffering from hematologic and non-hematologic disorders, but the application is limited by the identification of a suitable donor. Umbilical cord blood (UCB) is an alternative source of hematopoietic stem cell (HSC) transplantation. Despite all advantages, the limited cell dose is one of the  major obstacles. Ex-vivo expansion of HSC is an alternative way to overcome this problem.

**Materials and Methods: **In this study, polycaprolactone (PCL) scaffold coated with fibronectin (3D) is compared to routine cell culture system (two dimensional, 2D) used for cell culture.1×10^4^ cord blood CD34+ cells isolated by MACS were seeded on PCL scaffold and allowed to expand for 10  days. Before and after this period, total cells, CD34^+^ cells, CFC assay and CXCR4 expression were evaluated.

** Results: **Our findings demonstrated that 3D scaffold produced a 58-fold expansion of total cells compared to 2D cultures (38-fold expansion). Also CD34+ cells in 3D compare to 2D cell culture was 40-fold and 2.66 fold increased, respectively; this difference was statistically significant (p<0.05). Moreover, total number of colonies in the 3D scaffold was higher than those of 2D cell culture system, but no statistically significant difference was observed. Higher expression of CXCR4 in 3D compared to 2D showed better homing of cells that were cultured in 3D scaffold (p<0.05).

**Conclusion: **PCL scaffold coated with fibronectin had higher number of total cells and CD34+cells than 2D routine culture system. Findings revealed that 3D is a proper cell culture system for hematopoietic stem cell expansion, compared to 2D.

## Introduction

 Allogeneic hematopoietic stem cell (HSC) transplantation is used for treatment of several hematologic disorders and immune deficiencies, but the major obstacle to the use of this source is finding suitable donors for most patients.^   1 ^ Umbilical cord blood is rich in HSCs and is an alternative source for hematopoietic stem cell transplantation. Despite the advantages of umbilical cord blood such as lower incidence of GVHD after transplantation, little or no risk for donors, low risk of contamination, less HLA restriction and easy access, the major disadvantage remains delayed engraftment which results in delayed immune reconstitution and high rate of mortality, compared to BM source.    ^2^^-^^5^  Cell dose of cord blood is the most important factor for transplantation,^   6 ^ and to overcome this limitation several efficient strategies such as ex-vivo expansion by 3D scaffold,^   7 ^ co-culture by mesenchymal stem cell,^   8 ^ bioreactor^   9 ^ and etc., have been developed. Hematopoietic stem cells in bone marrow are in a specific place named “niche” cells.^   10 ^ Bone marrow established a balance between stem cell self-renewal and differentiation. It seems that the design of scaffold that mimics all the essential characteristics of BM  niche can maintain stem cells in a self-renewable state and influence the differentiation of stem cells. Moreover, interaction between stem cells and their niches can modulate HSC functions in vitro.^   11 ^ The fate of stem cells are affected by several factors such as hormones, cytokines, extracellular matrix (ESM)and cell interaction with other cells and tissues.^   12 ^ ECM component has a crucial role in HSC niche. Adhesiveness or interaction between HSCs and cell adhesion molecule providing homing or retaining HSCs in bone marrow niche are provided by these elements.^12^^,^^13^  Most of the in vitro cell culture systems currently used for expansion of hematopoietic stem cells are 2D systems.^   14 ^ It is important that 3D scaffold, producing sufficient cell number, retains the capacity for self-renewal and maintains proliferation of HSCs in cell culture medium.Fabrication of 3D scaffolds is one of the effective methods to promote cell growth and provide structural support.^   15 ^

 Today, different 3D scaffolds have been used for ex-vivo expansion of HSC including nanofiber mesh, porous matrices, woven and non-woven fabrics and microspheres. These fibers have different materials such as Polydimethylsiloxane (PDMS), poly-L-lactide acid (PLLA), Poly lactic-co-glycolic acid (PLGA), Polycaprolactone (PCL) and polyethylene terephthalate (PET).^   16 ^ The purpose of this study is to establish the new 3D culture system by using specific nanofiber and polycaprolactone (PCL) coated with fibronectin for ex-vivo expansion of cord blood hematopoietic stem cells. 

## MATERIALS AND METHODS

**Isolation of CB-CD34** ^+^ **progenitors**

 After obtaining informed consent, human cord blood was collected from donors. For isolation CD34+ cells, mononuclear cells (MNC) were separated by Ficoll-Hypaque gradient centrifugation (density 1.077 g/mL, Sigma) and then MACS CD34+ cell isolation kit was used (miltenyibiotec, USA***)***. Briefly, after centrifugation (Eppendorf) and cell counting, 300µl buffer was added up to 10^8^ total cells. Then, 100 µl blocker and 100 µl CD34 micro beads were added, mixed and incubated in 4^0C^ for 30 minutes. Cells were washed with buffer and centrifuged at 300×g for 10 minutes. Next, the supernatants were aspirated completely and resuspended in 500 µl buffer. MS column was placed in a magnetic field and rinsed with buffer. Cell suspension was then applied onto the column. The column was washed, removed from the separator and placed into 15ml falcon tube. Afterwards, the buffer was added to the column and then the magnetically labeled cells were immediately flushed out by firmly pushing the plunger into the column. culture medium was prepared by Stem line II serum-free media(Sigma, Germany) supplemented with recombinant human stem cell factor (SCF, 50 ng/mL, Peprotech, UK), recombinant human thrombopoetin (TPO, 50 ng/mL, Gibco, USA) and recombinant human FMS-like tyrosine kinas 3 (FLT-3, 50 ng/mL, Gibco, USA).

**Cell seeding into 3D scaffolds and control**

 PCL scaffolds were sterilized in 70% ethanol, washed by PBS and then dried overnight under sterile condition. Scaffolds were placed in 24-well polystyrene plates (Grainger) and coated with fibronectin in 50µg/ml concentration for 24h at 4^0C^. Then, we removed fibronectin solution and seeded cells. In each well, 1×10^4^CD34+ cells suspended in 250 µl culture medium were added. Plate was incubated at 37^0C^ in 5% Co2 for 10 days. Half of the medium was exchanged every 48h with fresh medium and cells were counted.

**Immunophenotype analysis**

 To evaluate the ability of HSC expansion and differentiation, cells were removed from scaffold and then the morphology of cells was checked and stained with monoclonal antibody CD34-PE and CD45-FITC (Stem cell technology, Canada) against human epitope. The tubes were incubated in 4^0C^ for 30 minutes. Isotype control was used to set compensation and confirm the specificity. 10000 events were acquired on a Partec PAS flow cytometer. Flow Jo software was used for data analysis.

**Colony assay**

 After 10 days of expansion, 10000 of expanded cells were harvested and transferred to 2 ml of methylcellulose medium (H4435, stem cell technology, Canada) plated in 6-well dishes (Grainger). H4435 contains 1% methylcellulose in Iscove’s MDM (IMDM, sigma, Germany). After 14 days incubation in 37^0C^ and 5% CO2, CFU-GM, BFU-E/CFU-E and CFU-GEMM (CFU-Mix) colonies were counted. 

**Gene expression**

 To evaluate the homing ability of expanded cells before and after expansion, CXCR4 expression was assessed by qPCR. RNA isolated by Trizol (Sigma, Germany) protocol. Briefly, 1ml Trizol was added and mixed well with every 10^6^ cells; then 200µl chloroform was added and centrifuged at 4^0C^ for 15 minutes in 12000 rpm. The aqueous phase was transferred to a new tube; 500µl isopropanol was added and centrifuged at 4^0C^ for 10 minutes in 12000 rpm. The supernatant was then removed and only RNA plate was washed by 75% ethanol for 5 minutes in 4^0C^ for 7500 rpm. The supernatant was then removed and 20µl DEPC water was added to RNA. To evaluate RNA quality, the value of OD_260/280_ and the concentration of RNA were measured using Nano Drop (Nano Drop 2000c) system. cDNA synthesis was performed by Thermo Scientific kit. For this purpose, 200 ng total RNA was mixed with 1µl oligo dt primer, 0.5 µl AMV enzyme, 4 µl buffer 4X, 0.5 µl RNAse inhibitor and 1 µl dNTP in tube and then nuclease free water was added up to a final volume of 20 µl. After mixing well, the tube was placed in a thermocycler using the following program: 10 min in 25 ^0C^, 1h in 42^0C^ and 10 min 75^0C^. After synthesis of cDNA, 1 µl cDNA was added and mixed by real-time master mix (Ampilicon, Denmark), gene-specific primers for CXCR4 and GAPDH control gene (Table 1). It was then watered up to 10 µl and placed in real-time PCR (ABI, USA) in the following program: 15 in 95 ^0C^ (1 cycle), 20 sec in 95 ^0C^ and 60 sec in 60 ^0C^ (40 cycles).

**Statistical analysis**

 Statistical analysis performed by T-test using Graph Pad prism6 software. Also, Real time PCR data was analyzed by REST.2009 software. 

## Results

**Flowcytometry analysis**

**Figure 1:**
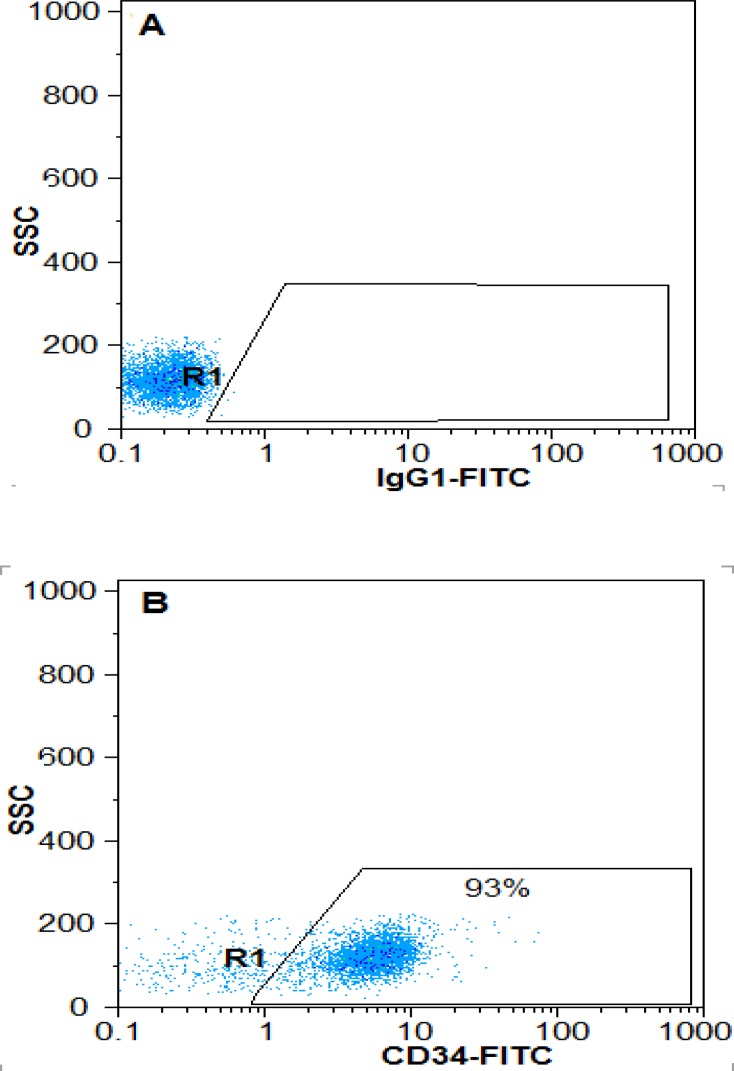
Purity of CD34+ cells before expansion. After isolation of cord blood cells, the surface marker CD34 was assessed immediately. A) Isotypecontrol, B) Percentage of CD34+ cells from MNC isolated cord blood

**Figure 2:**
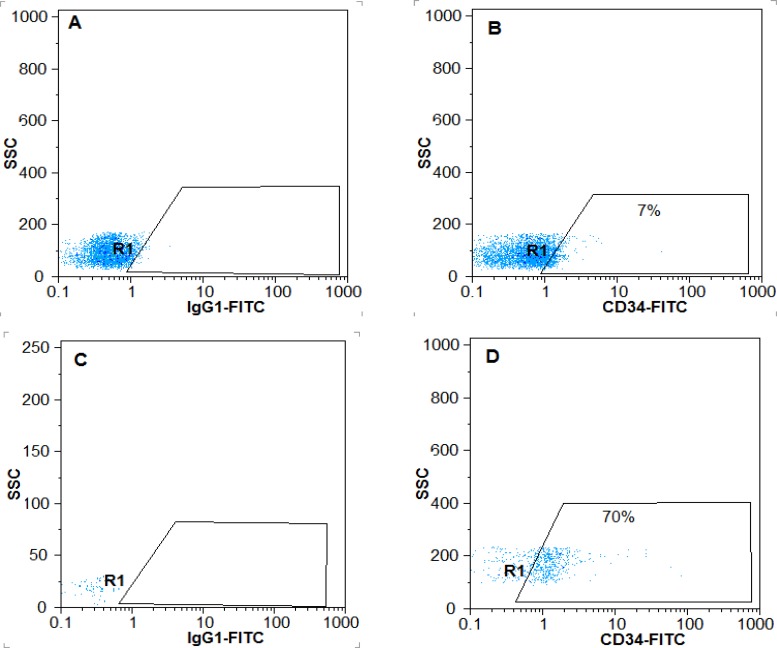
Percentage of CD34 + cells after a10-days extension on scaffold PCL nanofibers coated with fibronectin and the two-dimensional environment. A) Isotype control in 2Denvironment, B) The percentage of CD34 + cells in the two-dimensional environment, C) Isotype control in3-D environment, D) The percentage of CD34 + cells in three-dimensional environment.

 The purity of CD34+ cell cells isolated from cord blood before and after expansion in 3D scaffold was 93% (Figure1) and 65.66±5.35%, respectively. It was reported 7±0.86% in the control group, showing statically significant difference (Figure2) (p<0.05).

**Expansion of cultured cells in 3D PCL coated with fibronectin microenvironment**

**Figure 3:**
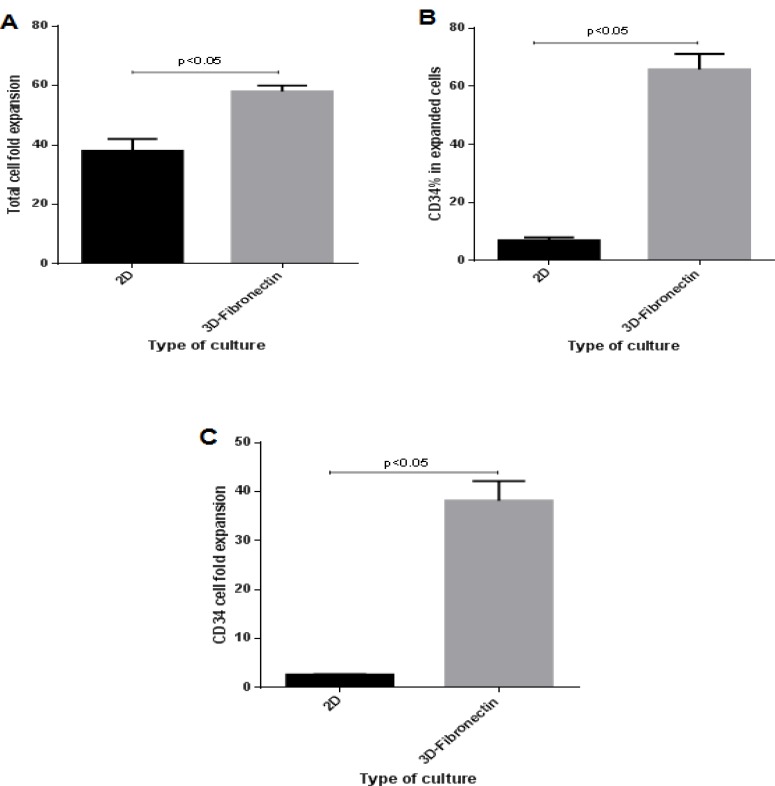
Effects of culture system on conventional and PCL nanofibers scaffold coated with fibronectin (2D versus 3D) on expansion of hematopoietic stem cells. 10000 cells of CD34 ^+^ cord blood (93%) culture on the conventional and scaffolds for 10 days in serum-free culture medium and analysis of the environment were taken. A) Total cell fold expansion, B) Percentage of CD34^+^, C): CD34+ cells fold expansion.

 After a 10-day expansion culture in serum-free medium, CD34^+^CB cells that cultured in 2D culture wells showed 38±3.3 fold expansion of total cells and 2.6±0.23 fold expansion of CD34^+^ cells (Figure 3). Fibronectin-coated PCL scaffold resulted in significant improvement in expansion of CD34^+^ cells with 58±1.63 fold expansion of total cells and 38.8±1.79 fold expansion of CD34^+^ cells (p <0.05).

**Colony assay**

**Figure 4:**
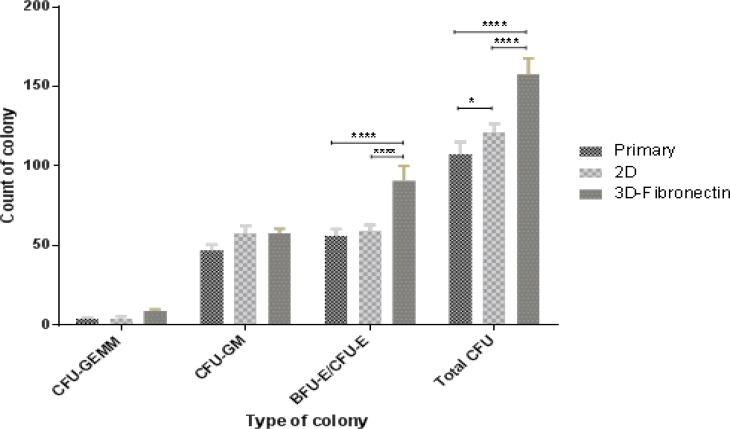
CFU numbers and progenitor percentage (CFU-GM, BFU-E/CFU-E, CFU-GEMM) based on total CFU analyzed for cells expanded in different culture system for 10 days

 To quantitate the clonogenic potential of hematopoietic stem cells and progenitors in the expanded cells, CFU assays were performed on cells expanded under PLC coated with fibronectin scaffold and plastic dish culture conditions (2D) before and after 10 days of culture. The BFU-E/CFU-E count in 3D scaffold, 2D culture system and primary cells was 90.67±7.76, 59±3.27 and 53±7.78, respectively. These differences were statistically significant (<0.05). The CFU-GM count in 3D scaffold (58±2.16) was higher than primary (47±2.94) and 2D culture system (54.33±8.73), but these differences were not statistically significant (p>0.05). CFU-GEMM colony counts in all cell culture systems were not statically significant (p>0.05). Also, total colonies in 3D and 2D culture systems were higher than primary cell culture (157.67±8.18, 121±4.55 and 107.67±6.13, respectively) and 3D was higher than 2D cell culture system, showing statically significant difference (p<0.05) (Figure 4).

**Evaluation of HSC homing**

**Figure 5:**
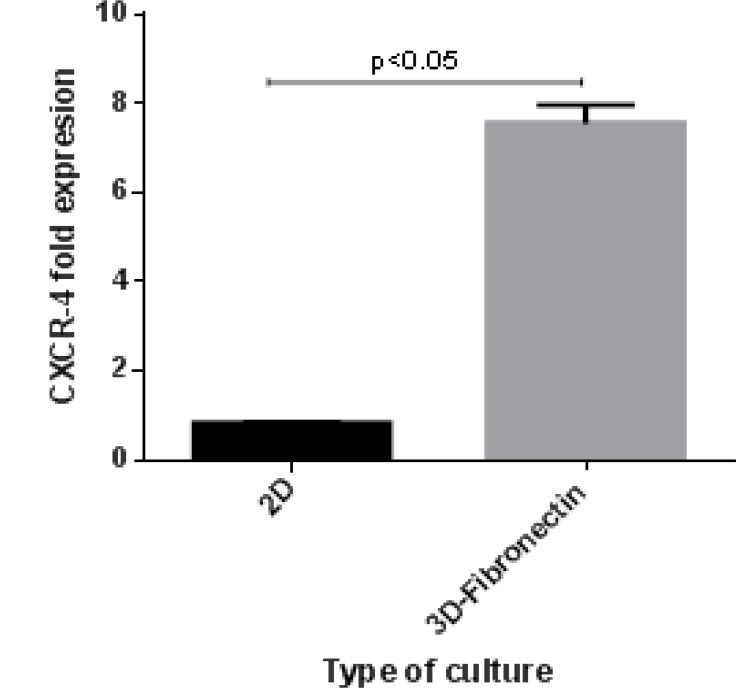
The changes in the expression of CXCR-4 after 10 days culture in 3D compare to 2D

 To evaluate homing and migration of expanded cells, assessment of homing factors such as CXCR4 can show the status of homing. CXCR-4 relative expression in 3D scaffold and in 2D cell culture system compared to before cell culture system had increased 7.2 fold and decreased -0.8 fold, respectively. CXCR-4 relative expression in 3D scaffold increased 8.6 fold compared to 2D culture (Figure 5).

## Discussion

 By the first successful UCBT and thereinafter establishment of cord blood banks, UCB was increasingly used in the treatment of hematologic and non-hematologic disorders. UCB is a valuable source of HSCT which can be used as an alternative for hematopoietic stem cell transplantation, as they are easily accessible and more readily available when a suitable donor is unavailable. Despite all advantages, the limited cell dose is one of the  major obstacles. One of the best ways to overcome this problem is expansion of HSCs focusing on maintaining self-renewability and stemness.

 In this research, 3D scaffold coated with fibronectin was used as it had a higher capacity for HSC expansion and lower differentiation compared to 2D cell culture system. Moreover, flow cytometric analysis demonstrated lower differentiation in 3D scaffold compared to the control group. The cause of this difference improves the interaction of cells and mimicking of physiochemical, cellular and natural microenvironment (niche) in 3D scaffold. 3D compartment is used to elevate the surface- to-volume ratio to increase cell interaction. 

 In 2D cell culture system, this interaction capacity is low and limited between neighbor cells.^   18 ^ In another study, different scaffolds with variable coating material were used for cord blood expansion. Ehring et al. showed that cytomatrix (tantalum-coated porous biomaterial) system produced a great number of CD34+ cells compared to 2D cell culture and culture in presence of bone-marrow stromal cells. Also, CD34+ and CD45+ increased 3-fold in cytomatrix and 1.3 and 1.6 increase in 2D cell culture in analysis by flowcytometry, respectively. ^19^  Ferreira et al.(2012) demonstrated that 3D scaffolds (PCL, fibrin and collagen) with and without co-culture of cord blood mesenchymal stem cells had large number of CD34+ cells compared to 2D cell culture microenvironment.^   15 ^ In another study, Feng et al. showed that Fibronectin-conjugated PET film resulted in a significant increase of CD34+ and total cell expansion compared to 2D cell culture.^   20 ^ Also, our results support  the findings of previous studies and showed that 3D scaffold system had a better environment for expansion of CD34+ and total cells compared to 2D cell culture system. Our results showed that total cells and CD34^+^ cells in 3D scaffold were increased by 58 and 38-fold, respectively. 

Colony-forming potential depended on number of hematopoietic stem cells and progenitor cells. According to higher number of HSCs in 3D scaffold, it seems that total colonies in 3D culture system were higher than those of 2D culture system. Ehring et al. (2003) showed the CFU-GM count in 3D culture system was higher than routinely cell culture system. Another study demonstrated that scaffolds conjugated with fibronectin and collagen had more total colonies than 2D cell culture system but this difference was not statistically significant.^   19 ^ Our results in CFC assay were similar to the above-mentioned studies. In 3D culture system, the total number of colonies was higher than that of 2D cell culture system, but no statistically significant difference was found. Also, BFU-E/CFU-E colony numbers were higher in 3D when compared with those of 2D cell culture, but CFU-GM colony numbers were lower in 3D than 2D.

 Expression of genes involving in homing of HSCs allows them to be placed in proper site. But, cell culture system may change their properties. The culture systems that can maintain the potential and homing properties of HSCs such as BM are better than others. Therefore, in this study, we evaluated CXCR4 expression in 3D culture system.

Ehring et al. reported that this epitope was expressed on a large percentage of CD34 cells in cytomatrix cell culture model.^   19 ^ Our results demonstrated a 5.5-fold increase in CXCR4 expression in PCL scaffold coated with fibronectin compared to 2D cell culture model.

## CONCLUSION

 In this study, we found that 3D PCL scaffolds coated by fibronectin were ideally suited for the expansion of HSC. The use of this scaffold resulted in higher cell proliferation rates, increased adhesion and better homing cell.

**Table 1:** Primer sequence of genes

**Genes**	**Primer** **sequence**
CXCR-4 F	TGAACCCCATCCTCTATGCTT
CXCR-4 R	GATGAATGTCCACCTCGCTTT
GAPDH-F	TGCACCACCAACTGCTTAGC
GAPDH-R	GGCATGGACTGTGGTCATGAG

## References

[1] Rocha V, Wagner Jr JE, Sobocinski KA (2000). Graft-versus-host disease in children who have received a cord-blood or bone marrow transplant from an HLA-identical sibling. New England Journal of Medicine.

[2] Barker JN, Krepski TP, DeFor TE (2002). Searching for unrelated donor hematopoietic stem cells: availability and speed of umbilical cord blood versus bone marrow. Biology of Blood and Marrow Transplantation.

[3] Dalle J, Duval M, Moghrabi A (2004). Results of an unrelated transplant search strategy using partially HLA-mismatched cord blood as an immediate alternative to HLA-matched bone marrow. Bone marrow transplantation.

[4] Davey S, Armitage S, Rocha V (2004). The London Cord Blood Bank: analysis of banking and transplantation outcome. British journal of haematology.

[5] Zarrabi M, Mousavi SH, Abroun S (2014). Potential uses for cord blood mesenchymal stem cells. Cell Journal (Yakhteh).

[6] Stanevsky A, Goldstein G, Nagler A (2009). Umbilical cord blood transplantation: pros, cons and beyond. Blood reviews.

[7] Sachlos E, Czernuszka J (2003). Making tissue engineering scaffolds work. Review: the application of solid freeform fabrication technology to the production of tissue engineering scaffolds. Eur Cell Mater.

[8] Robinson S, Ng J, Niu T (2006). Superior ex vivo cord blood expansion following co-culture with bone marrow-derived mesenchymal stem cells. Bone marrow transplantation.

[9] Cabral J (2001). Ex vivo expansion of hematopoietic stem cells in bioreactors. Biotechnology Letters.

[10] Wilson A, Trumpp A (2006). Bone-marrow haematopoietic-stem-cell niches. Nature Reviews Immunology.

[11] Wilson A, Oser GM, Jaworski M (2007). Dormant and Self‐Renewing Hematopoietic Stem Cells and Their Niches. Annals of the New York Academy of Sciences.

[12] Liu H, Lin J, Roy K (2006). Effect of 3D scaffold and dynamic culture condition on the global gene expression profile of mouse embryonic stem cells. Biomaterials.

[13] Vazin T, Schaffer DV (2010). Engineering strategies to emulate the stem cell niche. Trends in biotechnology.

[14] Even-Ram S, Yamada KM (2005). Cell migration in 3D matrix. Current opinion in cell biology.

[15] Ventura Ferreira MS, Jahnen-Dechent W, Labude N (2012). Cord blood-hematopoietic stem cell expansion in 3D fibrin scaffolds with stromal support. Biomaterials.

[16] Hu J, Ma PX (2011). Nano-fibrous tissue engineering scaffolds capable of growth factor delivery. Pharmaceutical research..

[17] Gluckman E, Rocha V (2005). History of the clinical use of umbilical cord blood hematopoietic cells. Cytotherapy.

[18] Dhandayuthapani B, Yoshida Y, Maekawa T (2011). Polymeric scaffolds in tissue engineering application: a review. International Journal of Polymer Science.

[19] Ehring B, Biber K, Upton T (2003). Expansion of HPCs from cord blood in a novel 3D matrix. Cytotherapy.

[20] Feng Q, Chai C, Jiang XS (2006). Expansion of engrafting human hematopoietic stem/progenitor cells in three‐dimensional scaffolds with surface‐immobilized fibronectin. Journal of Biomedical Materials Research Part A.

